# Extracellular histones trigger oxidative stress-dependent induction of the NF-kB/CAM pathway via TLR4 in endothelial cells

**DOI:** 10.1007/s13105-022-00935-z

**Published:** 2022-12-05

**Authors:** Daniel Pérez-Cremades, Carlos Bueno-Betí, José Luis García-Giménez, José Santiago Ibañez-Cabellos, Federico V. Pallardó, Carlos Hermenegildo, Susana Novella

**Affiliations:** 1 Department of Physiology, Faculty of Medicine and Dentistry, University of Valencia, Av/Blasco Ibañez, 15, 46010 Valencia, Spain; 2 INCLIVA Biomedical Research Institute, Valencia, Spain; 3 Center for Biomedical Network Research On Rare Diseases (CIBERER), Institute of Health Carlos III, Valencia, Spain

**Keywords:** Endothelium, Extracellular histones, Inflammation

## Abstract

Extracellular histones have been reported to aggravate different pathophysiological processes by increasing vascular permeability, coagulopathy, and inflammation. In the present study, we elucidate how extracellular histones (10–100 µg/mL) concentration dependently increase cytosolic reactive oxygen species (ROS) production using human umbilical vein endothelial cells (HUVECs). Furthermore, we identify cyclooxygenase (COX) and NADPH oxidase (NOX) activity as sources of ROS production in extracellular histone-treated HUVEC. This COX/NOX-mediated ROS production is also involved in enhanced NF-kB activity and cell adhesion molecules (VCAM1 and ICAM1) expression in histone-treated HUVEC. Finally, by using different toll-like receptor (TLR) antagonists, we demonstrate the role of TLR4 in CAMs overexpression triggered by extracellular histones in endothelial cells. In conclusion, our data suggest that through TLR4 signaling, extracellular histones increase endothelial cell activation, a mechanism involving increased COX- and NOX-mediated ROS. These findings increase our understanding on how extracellular histones enhance systemic inflammatory responses in diseases in which histone release occurs as part of the pathological processes.

**Supplementary Information:**

The online version contains supplementary material available at 10.1007/s13105-022-00935-z.

## Introduction

Histones are essential proteins regulating chromatin conformation and gene transcription, but when histones are released into extracellular space, they can mediate proinflammatory activity [3]. Extracellular histones act as an aggravating factor in multiple pathophysiological processes and disease progression [7]. Among these, they have been implicated in organ injury after trauma [1], autoimmune diseases [21], and ischemic heart disease [5]. In addition, when released into the bloodstream, histones mediate in the pathology of strokes [9], disseminated intravascular coagulation [23], sepsis [32], and septic shock [11].

As a communicative dynamic barrier between intravascular and extravascular spaces, endothelium is responsive to circulating compounds, including extracellular histones. Indeed, it has been reported that organ injury mediated by extracellular histones is caused primarily through endothelial damage [18] and induced endothelial barrier dysfunction [12]. Specifically, molecular studies in histone-exposed endothelial cells have pinpointed their roles in inducing Ca^2+^ overload [8], increasing cell adhesion molecules (CAMs) [35] and tissue factor expression [34], and disarranging vasoactive mediator release [25], thus altering vascular homeostasis. In addition, both apoptotic and autophagy pathway activation have been implicated in histone-mediated endothelial cell death [16].

Dying cells and neutrophil extracellular trap (NET) formation by activated neutrophils have been shown to be major endogenous sources of extracellular histones [7, 10]. These histones and nucleosomes released into the bloodstream are described as damage-associated molecular patterns (DAMPs) [14] whose response is driven by pattern recognition receptors (PPRs) [4]. Among PPRs, toll-like receptors (TLRs) have been reported as the main receptors for extracellular histones [7]. In this regard, endothelium expresses different TLRs [31], which modify its physiology upon exposure to extracellular histones, ultimately resulting in endothelial injury and dysfunction [7].

Extracellular histones have been implicated in increased production of reactive oxygen species (ROS) [17, 27]. Production of ROS has been identified as a key component in progression of many inflammatory diseases, acting both as signaling molecule and inflammatory mediator [20]. Although recent data have provided insight into several actions triggered by extracellular histones in endothelial cells, the mechanisms by which extracellular histones increase ROS production remain unclear.

Although previous studies have reported that extracellular histones increase intracellular ROS levels in human umbilical vein endothelial cells (HUVEC) [25], the sources of ROS involved in histone-triggered effects have been less studies. Here, we aimed to get deeper insight into the mechanism involved in the production of oxidative stress in HUVEC exposed to extracellular histones, interrogating for the main oxidative stress generating cellular factors. Furthermore, we investigated the role of increased ROS production in the activity of key inflammatory modulator NF-kB, and expression of different CAMs in human endothelial cells exposed to extracellular histones. Finally, we determined the role of different TLR types in the observed histone-triggered response.

## Methods

### Cell culture and experimental design

Pooled human umbilical vein endothelial cells (HUVECs) from 5 individual female donors were purchased from Lonza (Barcelona, Spain) and were grown in Medium 199 (Sigma-Aldrich, Madrid, Spain) supplemented with 20% fetal bovine serum (Gibco, Invitrogen, Barcelona, Spain), endothelial cell growth supplement from bovine neural tissue (ECGS, Sigma-Aldrich), and heparin sodium salt from porcine intestinal mucosa (Sigma-Aldrich). Cells were routinely grown in an incubator at 37 °C with 5% CO_2_. HUVECs from passages 3 to 5 were used in this study. When they reached confluence, the media was changed and cells underwent 4 h exposure to different calf thymus histone concentrations (Sigma-Aldrich, St. Louis, MO, USA): 10, 25, 50, or 100 µg/mL prepared in PBS. In some experiments, 30 µM apocynin (Sigma-Aldrich), 10 µM indomethacin (Sigma-Aldrich), 10 µM celecoxib (Sigma-Aldrich), 100 µmol/l tempol (Sigma-Aldrich), 20 µM Bay11-7082 ((E)-3-(4-methylphenylsulfonyl)-2-propenenitrile; Sigma-Aldrich), 20 µg/mL oxPAPC (Invivogen, Toulouse, France), 0.7 µM iODN (inhibitory oligodeoxynucleotide with phosphorothioate backbone, Enzo Life Science, Farmingdale, USA), and 3 µM CLI-095 (Invivogen) were added to HUVEC 1 h prior to histone treatment.

### Reactive oxygen species production measurement

Intracellular reactive oxygen species (ROS) production was detected using fluorescence probes: dihydroethidium (DHE, Invitrogen) for intracellular ROS or MitoSOX (Invitrogen) for mitochondrial ROS. Histone-treated cells were loaded with 2.5 μM DHE or 5 μM MitoSOX for 30 min. Next, cells were rinsed with PBS and observed under an inverted fluorescence Nikon Eclipse Ti-S microscope. Fluorescence was measured from three different fields per well. Fluorescence signals were quantified using NIS-Elements 3.2 software (Nikon Izasa S.A, L’Hospitalet de Llobregat, Spain).

### Cell transfection

HUVECs were cultured overnight before being transfected with Lipofectamine 2000 transfection reagent (Thermo Fisher Scientific). Short interference (si)RNA negative control (Assay ID 117,432) and siRNA NOX1 inhibitor (Assay ID 4,390,843) were used at 20 nM in serum-free OptiMEM medium (Gibco) during 48 h. HUVEC transfected with siNOX1 showed ~ 40% reduction in NOX1 protein levels (Suppl. Figure 1B).

### Gene expression analysis by RT-qPCR

Total RNA was isolated from cells using NucleoSpin® RNA/Protein (740,933.50, Macherey–Nagel, Düren, Germany) according to the manufacturer’s instructions. For reverse transcription (RT) reactions, 200 ng of purified RNA was reverse transcribed using random hexamers with the high-capacity cDNA reverse transcription kit (P/N 4,322,171, Applied Biosystems, Foster City, USA) according to the manufacturer’s instructions.

Gene expression was determined by quantitative real-time PCR analysis using an ABI Prism 7900 HT Fast Real-Time PCR System (Applied Biosystems, Foster City, CA, USA). Gene-specific primer pairs and probes were purchased from Applied Biosystems (Assays-on-Demand) for SOD1 (Hs00533490_m1), SOD2 (Hs00167309_m1), VCAM1 (Hs01003372_m1), ICAM1 (Hs00164932_m1), and GAPDH (Hs99999905_m1), and were used together with TaqMan Universal PCR Master Mix (P/N 4,304,437) and reverse-transcribed sample RNA in 20-μl reaction volumes. iTaq TM Universal SYBR Green supermix (Bio-Rad Laboratories Inc., Madrid, Spain) was also used in order to determine mRNA expression. Primers used in SYBR green-based qRT-PCR were all purchased from Sigma-Aldrich. Primers sequences are described in Table 1. PCR conditions were determined according with manufacturer’s instructions. Glyceraldehyde-3-phosphate dehydrogenase expression levels were measured in all samples to normalize differences in RNA input, RNA quality, and reverse transcription efficiency. Each sample was analyzed in triplicate, and expression was calculated according to the 2^−ΔΔ*C*t^ method.

**Table 1:** Primer sequences

Gene	Sense (5′—3′)	Antisense (3′—5′)
GAPDH	TCGGAGTCAACGGATTTG	CAACAATATCCACTTTACCAGAG
IL18	CCTTTAAGGAAATGAATCCTCC	CATCTTATTATCATGTCCTGGG
IL1A	AGAGGAAGAAATCATCAAGC	TTATACTTTGATTGAGGGCG
IL1B	CTAAACAGATGAAGTGCTCC	GGTCATTCTCCTGGAAGG
MYD88	GTTGTCTCTGATGATTACCTG	GGGGAACTCTTTCTTCATTG
NOX1	CCGGTCATTCTTTATATCTGTG	CAACCTTGGTAATCACAACC
NOX2	AAGATCTACTTCTACTGGCTG	AGATGTTGTAGCTGAGGAAG
NOX4	AATTTAGATACCCACCCTCC	TCTGTGGAAAATTAGCTTGG

### Protein expression analyzed by Western blot

Proteins extracts (50 μg) were denatured with sample buffer (Tris 40 mM, EDTA, bromophenol blue 0.01%, sucrose 40%, SDS 4%, β-mercaptoethanol 10%) and heated to 95 °C for 5 min. Afterwards, samples were electrophoresed in 12% SDS-PAGE and transferred onto nitrocellulose membrane (Whatman GmbH, Dassel, Germany).

After transference, the membrane was blocked with 5% milk or 5% BSA (in the case of phosphorylated proteins) in Tris-buffered saline and Tween 20 (TBST) for 1 h. Afterwards, the membranes were incubated with the specific primary antibodies: p65 subunit of NF-κB, p-p65(Ser276), SOD1, and SOD2 (from Cell Signaling, Beverly, MA, USA); NLRP3 (from Novus, NBP2-12,446); COX1, COX-2, NOX1, NOX4, and β-actin as loading control (from Santa Cruz BioTech, Dallas, TX, USA).

Afterwards, the blots were washed again with TBST and incubated for a further 1 h with a secondary mouse, rabbit, or goat antibody with horseradish peroxidase-linked conjugate. The membrane was incubated at room temperature with constant agitation. Finally, the membrane was washed 3 × 5 min with TBST. Luminol was added onto the membrane (ECL Western Blotting Detection Reagents, GE Healthcare, Hatfield, and Hertfordshire, UK), and membrane chemiluminescence was revealed by LAS-4000 image reader (GE Healthcare).

### Statistical analysis

Values are expressed as mean ± standard error of mean (SEM). Student’s *t*-test was applied for between-group comparisons. One-way analysis of variance was used to determine the difference between groups. When an interaction effect was found, multiple comparisons were performed using the Student–Newman–Keuls method “post hoc” test. Statistical significance was set at *P < 0.05, **P < 0.01, and ***P < 0.001, as indicated in each case. GraphPad Prism v6.0 (GraphPad Software, San Diego, CA, USA) was used for statistical analysis and graphic representations.

## Results

### Extracellular histones induce ROS production in a concentration-dependent manner and increase antioxidant enzyme expression in HUVEC

**Fig. 1:**
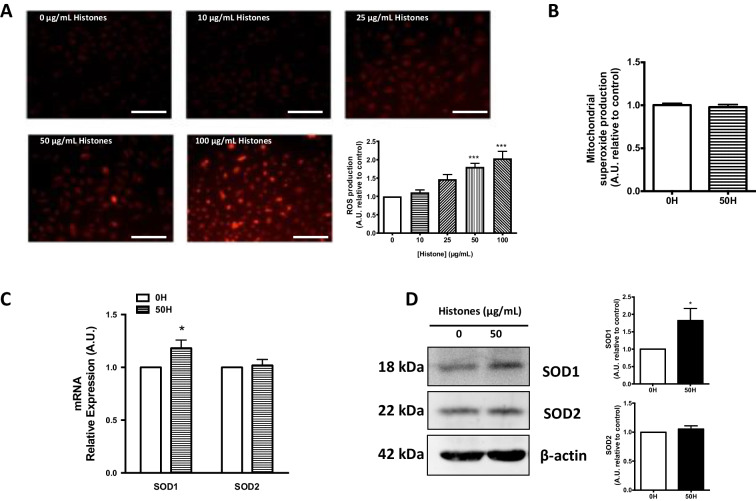
Extracellular histone-treated HUVEC increases ROS production and antioxidant response through increased SOD1 expression. **A** HUVECs were exposed to different concentrations of histones for 4 h and intracellular ROS levels were determined by DHE oxidation (*n* = 7) as described in Methods. **B** HUVECs were exposed to 0 µg/mL of histones (0H) and 50 µg/mL of histones (50H) for 4 h and mitochondrial ROS levels were determined by MitoSOX probe (*n* = 6) as described in Methods. **C** HUVECs were exposed to 50 µg/mL of histones for 4 h (*n* = 3). Relative SOD1 and SOD2 expression were determined by qRT-PCR. **D** Protein extracts (20 µg protein) from cultured HUVEC incubated at 50 µg/mL of histones for 4 h were loaded on SDS-PAGE gels and analyzed by Western blotting using anti-SOD1 and anti-SOD2. β-actin was used as loading control. One representative experiment of three performed is shown. Relative levels assessed by densitometry are presented. Data are expressed as mean ± SEM of *n* = 6–7. *P < 0.05 and ***P < 0.001 versus 0 µg/mL of histones

The first aim of the present work was to investigate the effect of extracellular histones on ROS production in endothelial cells. Exposure of HUVEC to increasing concentrations of extracellular histones (10, 25, 50, and 100 μg/mL) for 4 h resulted in a concentration-dependent increase of ROS production, showing statistically significance with concentrations above 50 μg/mL. Specifically, ROS production increased up to 80 ± 10% when cells were exposed to 50 μg/mL (*P* < 0.001), and up to 103 ± 19% when exposed to 100 μg/mL (*P* < 0.001) (Fig. 1A). Conversely, no changes in mitochondrial ROS production were observed in endothelial cells exposed to 50 μg/mL of extracellular histones compared to non-treated cells (Fig. 1B).

Furthermore, HUVEC exposed to extracellular histones (50 μg/mL) showed altered expression of cytosolic superoxide dismutase (SOD1), whereas no significant differences were observed for the mitochondrial variant SOD2. Results showed significantly increased SOD1 expression levels in HUVEC (21 ± 8%, *P* < 0.05; Fig. 1C) at 50 μg/mL of extracellular histones. These data were supported by protein levels: relative levels assessed by densitometry revealed a significant increase in the cytosolic SOD (82 ± 35%, *P* < 0.05), while conversely, mitochondrial SOD protein levels were found unaltered (Fig. 1D) in agreement to the mRNA levels measured by RT-qPCR.

### COX and NOX are involved in ROS production induced by extracellular histones in HUVEC

**Fig. 2:**
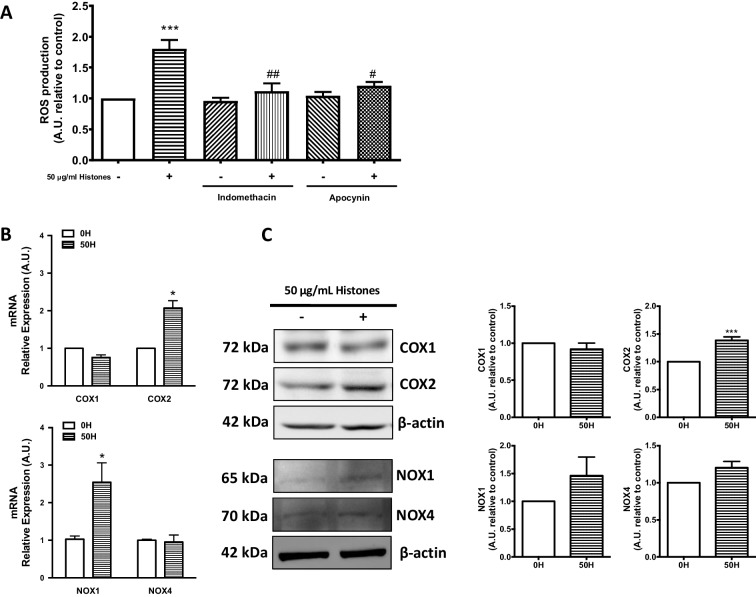
ROS induced by extracellular histones is mediated by COX and NOX activity. **A** HUVECs were preincubated with apocynin (Apo) and indomethacin (Indo) for 1 h and treated with 0 µg/mL of histones (0H) and 50 µg/mL of histones for 4 h (50H, *n* = 10). Intracellular ROS levels were determined by DHE oxidation as described in Methods. **B** HUVEC exposed to 50 µg/mL of histones for 4 h (*n* = 7). Relative COX-1, COX-2, NOX1, and NOX4 expression were determined by qRT-PCR. **C** Protein extracts (20 µg protein) from cultured HUVEC incubated at 50 µg/mL of histones for 4 h (*n* = 4) were loaded on SDS-PAGE gels and analyzed by Western blotting using anti-COX-1, anti-COX-2, anti-NOX1, and anti-NOX4. β-actin was used as loading control. One representative experiment of three performed is shown. Relative levels assessed by densitometry are presented. Data are expressed as mean ± SEM. *P < 0.05 and ***P < 0.001 versus 0 µg/mL of histones

We next sought to determine which specific sources of ROS production were induced in endothelial cells exposed to extracellular histones. NADPH-oxidase (NOX) [6] and cyclooxygenase (COX) [26] have been suggested as important sources of ROS production under inflammatory conditions. To evaluate the source of ROS production of histone-treated HUVEC, we incubated HUVEC with apocynin (antioxidant) and indomethacin (COX inhibitor) before extracellular histone (50 μg/mL) treatment. Results showed a significant decrease in ROS production when endothelial cells were treated with both apocynin and indomethacin. (Fig. 2A). In addition, to determine the role of extracellular histones in modulation of different isoforms of COX and NOX, HUVECs were treated with 50 μg/mL extracellular histones, and COX-1, COX-2, NOX1, and NOX4 mRNA expression and protein levels were determined. Histone-exposed HUVEC showed increased mRNA expression of COX-2 and NOX1, while COX-1 and NOX4 expression remained unaltered (Fig. 2B). Moreover, histones induced an increase in COX-2 protein production (38 ± 6%, *P* < 0.001) while COX-1 levels were unaltered. Conversely, no significant changes on NOX1 and NOX4 were observed (Fig. 2C). Altogether, these results suggest a role for COX and NOX enzymes in histone-dependent ROS production in HUVEC.

### ROS productions mediated by COX and NOX are involved in NF-kB/CAM pathway expression in histone-treated HUVEC

Given that increased ROS levels are associated with increased activity of pro-inflammatory mediators and with the expression of adhesion molecules, we next performed Western blot analysis to evaluate the levels and activity of NF-κB, a pivotal molecule in endothelial inflammation control, and the expression and activity of VCAM1 and ICAM1, both involved in leukocyte adhesion to endothelium monolayer.

**Fig. 3:**
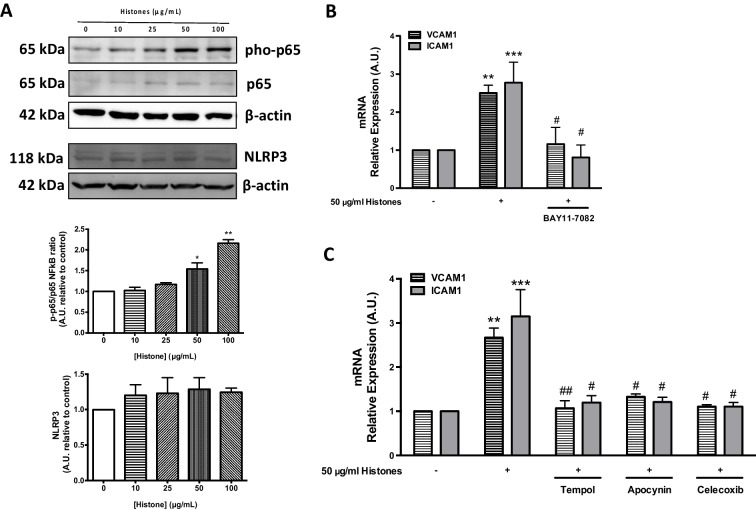
COX/NOX activity, via ROS production, is involved in NFkB-dependent VCAM1 and ICAM1 expression increase in extracellular histone-treated HUVEC. **A** Protein extracts (20 µg protein) from HUVEC exposed to different concentrations of histones for 4 h (*n* = 4) were loaded on SDS-PAGE gels and analyzed by Western blotting using anti-p-p65, anti-p65, and anti-NLRP3. β-actin was used as loading control. One representative experiment of three performed is shown. Relative levels assessed by densitometry are presented. **B** HUVECs were incubated with Bay11-7082 (Bay) and exposed to 50 µg/mL of histones for 4 h (*n* = 4). Relative VCAM1 and ICAM1 expression were determined by qRT-PCR. **C** HUVECs were incubated with tempol, apocynin, and celecoxib, and exposed to 50 µg/mL of histones for 4 h (*n* = 3). Relative VCAM1 and ICAM1 expression were determined by qRT-PCR. Data are expressed as mean ± SEM. *P < 0.05, **P < 0.01, and ***P < 0.001 versus 0 µg/mL of histones. ^#^*P* < 0.05, ^##^*P* < 0.01 versus 50 µg/mL of histones

Figure 3A shows a concentration-dependent increase of the p65 subunit of NF-κB and its phosphorylation in HUVEC treated with extracellular histones. Blot quantification revealed a significant increase of p65 phosphorylation when endothelial cells were exposed to 50 (*P* < 0.05) and 100 μg/ml (*P* < 0.01) of extracellular histones (Fig. 3A). Importantly, this effect was abrogated when cells were previously exposed to BAY11-7082, an inhibitor of p65 activation (Fig. 3B). Since Bay 11–7082 also acts as a selective inhibitor for nod-like receptor family pyrin domain containing 3 (NLRP3), we also determined the NLRP3 protein levels. However, no significant changes were observed in HUVEC exposed to increasing concentrations of extracellular histones (Fig. 3B). Also, no changes were observed in the expression of the pro-inflammatory cytokines IL-1β, IL-18, and IL-1α associated with NLRP3 in histone-treated cells (Suppl. Figure 1A).

To investigate the effect of extracellular histones in VCAM1 and ICAM1 expression, we determined mRNA expression in HUVEC by qRT-PCR. Histone-treated HUVEC showed significant increased expression (Fig. 3B) of both VCAM1 (150 ± 20%, *P* < 0.01) and ICAM1 (177 ± 53%, *P* < 0.001) at 50 μg/mL of histones. As occurred for p65 activation, BAY11-7082 caused a significant reduction in histone-mediated VCAM1 (*P* < 0.05) and ICAM1 (*P* < 0.05) mRNA levels (Fig. 3C), indicating that NF-κB is involved in adhesion molecule expression induced by extracellular histones in HUVEC.

In order to determine whether extracellular histone-induced ROS-generating enzymes are related to the observed pro-inflammatory pathway induction, we studied VCAM1 and ICAM1 mRNA expression in histone-treated HUVEC previously incubated with the superoxide dismutase mimetic tempol, apocynin, and the COX-2-specific inhibitor celecoxib. All treatments abrogated the VCAM1 and ICAM1 mRNA induction observed in endothelial cells exposed to extracellular histones (Fig. 3D). Moreover, specific siRNA knock-down of NOX-1 significantly reduced ROS production and VCAM1 expression in histone-treated cells (Suppl. Figure 1B). These results thus indicate the involvement of ROS, NOX-1 and COX-2, in the enhanced CAM expression observed in endothelial cells after extracellular histone exposure.

### TLR4 is involved in ROS-dependent CAM expression in extracellular histone-treated HUVEC

**Fig. 4:**
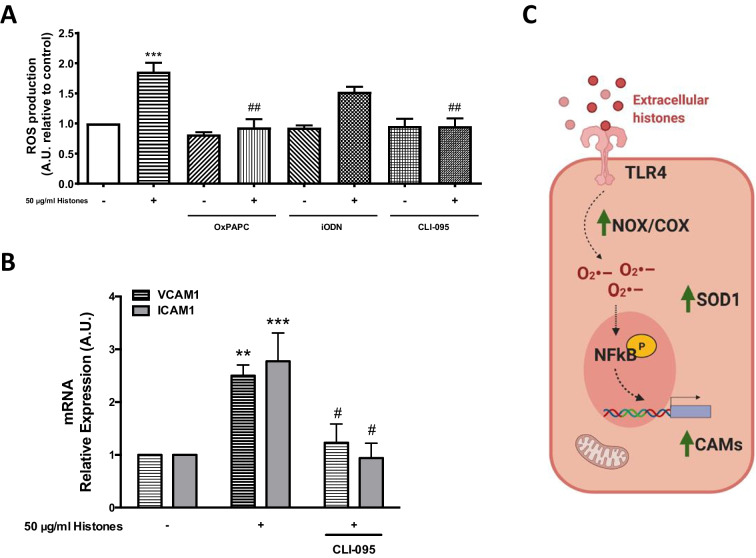
TLR4 is involved in ROS-dependent increase in VCAM1 and ICAM1 expression in histone-treated endothelial cells. HUVECs were preincubated with OxPAPC, iODN, and CLI-095 for 1 h and treated with 50 µg/mL of histones for 4 h (50H, *n* = 8). Intracellular ROS productions were determined by DHE oxidation as described in Methods. **B** HUVECs were incubated with CLI-095 and exposed to 50 µg/mL of histones for 4 h (*n* = 8). Relative VCAM1 and ICAM1 expression were determined by qRT-PCR. Data are expressed as mean ± SEM. **P < 0.01 and ***P < 0.001 versus 0 µg/mL of histones. ^#^*P* < 0.05, ^##^*P* < 0.01 versus 50 µg/mL of histones. **C** Working model

As described in the introduction, extracellular histones have been reported to bind to the cell surface through TLR receptors, although the specific TLR responsible for this process is still under debate [14, 33]. We found that preincubation of HUVEC with TLR inhibitors after extracellular histone treatment modulated ROS production (Fig. 4A). The results demonstrated that histone-induced ROS production decreased significantly upon preincubation with OxPAPC (a TLR2 and TLR4 inhibitor), but not when HUVECs were pre-treated with iODN (an TLR7 and TLR9 inhibitor). CLI-095, a selective TLR4 inhibitor, was used to determine the specificity of extracellular histone binding to this receptor in HUVEC. CLI-095 decreased histone-mediated ROS production in HUVEC (*P* < 0.01, CLI-095 + 50 μg/mL of histones relative to 50 μg/mL of histones) (Fig. 4A). In addition, expression of MYD88, an adapter protein that mediates signal transduction from TLRs to NF-κB, showed a trend for increased expression in HUVEC exposed to extracellular histones (50 μg/ml) compared to non-treated cells (Suppl. Figure 1C). Altogether, these results indicate that HUVECs exposed to extracellular histones exhibit an increase in ROS production via TLR4.

We further analyzed the role of TLR4 in CAM expression in histone-treated HUVEC. Extracellular histone-treated endothelial cells were preincubated with CLI-095 and VCAM1 and ICAM1 mRNA expression (Fig. 4B) were determined. Results showed reduced VCAM1 and ICAM1 mRNA expression in histone-incubated cells pre-treated with TLR4 antagonist, thus reverting the induction produced by 50 µg/mL of histones.

Taken together, our experiments indicate that extracellular histones increase cytosolic oxidative status in HUVEC, increasing ROS production and altering antioxidant enzymes. ROSs are produced by COX and NOX enzymes after extracellular histone exposure via a TLR4-dependent mechanism, which in turn leads to heightened NF-kB activation and VCAM1 and ICAM1 expression (Fig. 4C).

## Discussion

In this study, we demonstrated that endothelial cells exposed to extracellular histones enhance ROS production through a TLR4-COX/NOX pathway, which in turn increases cell adhesion molecules (VCAM1 and ICAM1) expression via NFkB activity. First, histone-treated HUVEC showed a concentration-dependent rise in cytosolic ROS production and a concomitant increase in the antioxidant cytosolic superoxide dismutase enzyme SOD1. Second, extracellular histone-induced superoxide anion production is mediated by COX and NOX activity. Third, COX/NOX-mediated increase in ROS induced VCAM1 and ICAM1 expression through an NFkB-dependent mechanism. Finally, we identified TLR4 as the main receptor involved in the above described pathway.

Extracellular histones contribute to the pathobiology of systemic inflammatory diseases in which endothelium activation seems to play a crucial, including both infections, such as sepsis [32], and sterile inflammation, such as stroke [9], disseminated intravascular coagulation [23], or ischemia–reperfusion injury [14]. Endothelial cells exposed to extracellular histones release proinflammatory cytokines [12], induce tissue factor expression [34], and show increased adhesion molecules in the cell membrane [35]. Indeed, in vivo experiments have demonstrated that administration of extracellular histone causes neutrophil migration, endothelial dysfunction, and thrombosis [32].

Our results demonstrate that extracellular histones increase ROS production in a concentration-dependent manner in endothelial cells. Production of ROS is vital in the pathogenesis of vascular injury and contributes to different vascular responses in inflammation, such as vasomotor dysfunction, impaired vascular permeability, enhanced thrombus formation, and leukocyte recruitment [19]. Increased ROS levels have also been observed in histone-treated cardiomyocytes [17] and Kuppfer cells [15]. Moreover, pretreatment of dendritic cells with antioxidants prevented H4-induced cytokine secretion [2]. Furthermore, histone-exposed endothelial cells showed increased expression of the cytosolic SOD1, which can be explained as an adaptive compensatory antioxidant mechanism in response to oxidative stress to maintain the redox-state balance [13].

Our results showed that histone-mediated ROS production depends on NOX and COX activity. In this regard, NOX-dependent overproduction of ROS observed in cardiac myocytes exposed to plasma from patients with sepsis [30] could be due to elevated circulating levels of pro-inflammatory mediators, including extracellular histones [11, 32]. Extracellular histones treatment of HUVEC did not change NOXs protein levels. In this regard, direct interaction of TLR4 with Nox4 has been reported as the mechanism involved in LPS-mediated ROS generation [24]. Furthermore, it has been reported that ROS production by NOX can subsequently trigger other ROS-generating sources [6] such as COX. Our findings indicate that extracellular histone treatment enhanced COX-2 expression while COX1 remained unaltered. In this regard, increased COX-2 expression has previously been observed in dermal microvascular endothelial cells exposed to *P. falciparum* histones [12]. As demonstrated by inhibiting COX-2 activity, ROS production should be mediated by COX-2 expression enhancement observed in histone-treated endothelial cells. In agreement with these results, multiple studies have focused on the contribution of COX-2-dependent oxidative stress in endothelial inflammation [29], suggesting its role as an inflammatory signal mediator.

The endothelium responds to inflammatory mediators by expressing adhesion molecules on the cell surface, increasing rolling, adherence, and transmigration of leukocytes into the underlying tissue. Here, we demonstrate elevated VCAM1 and ICAM1 expression in endothelial cells exposed to extracellular histones, which are known to contribute to inflammatory cell recruitment. These results agree with the previous findings of Shrestha et al. [27] and are in agreement with the results that histone-neutralizing antibodies significantly reduced neutrophil recruitment in an in vivo mice model of sterile inflammation [2]. Additionally, we show that the VCAM1 increased expression in histone-treated endothelial cells is dependent on NF-kB, a key inflammatory modulator whose activity can be regulated by the cellular redox status [22], in a concentration-dependent manner. Similar results have been shown using primary human coronary artery endothelial cells exposed to extracellular histones [34].

Our experiments using different TLR antagonists indicate that intervention on TLR4 can restore endothelial levels of ROS production enhanced by extracellular histone exposure, and hence levels of adhesion molecules in HUVEC. Several studies propose that extracellular histone action is triggered via TLRs [14, 33, 34], and our results reinforce this idea and further demonstrate that endothelial adhesion factors are stimulated via TLR4 in HUVEC. Extracellular histone release has been implicated in tissue factor expression in vascular endothelial cells via TLR2/4-dependent mechanisms [34]. In the liver, however, histone-induced tissue injury has been linked to activation of both TLR2/4 [33] and TLR9 [14]. Indeed, cell lineage could also be involved in TLR-mediated histone action, since it has been observed that extracellular histone-activated TLR9 leads to ROS production in Kupffer cells [15]. Furthermore, using KO mice, Xu et al. found that both TLR2 and TLR4 were implicated in histone-mediated cell death, but only TLR4 was responsible for histone-dependent increase of cytokines levels [33]. These results suggest that histone binding to specific TLRs could activate different molecular pathways that will result in a determinant response. Opposite to our results, histone-induced expression of adhesion molecules was inhibited by neutralizing antibodies anti-TLR9, but not by anti-TLR2 or anti-TLR4, suggesting that TLR9 is involved in the histone-induced induction of adhesion molecules in EA.hy926 endothelial cells [35]. Discrepancies could be due to the previously reported differences between the cell line EA.hy926 and HUVECs [28] and reinforce the idea that cell specificity may be related to TLR-mediated histone action.

In conclusion, our findings demonstrate that NOX and COX have a central role in enhanced ROS production exhibited in human endothelial cells exposed to extracellular histones. Furthermore, over-production of ROS in histone-treated HUVEC increases CAM expression in an NF-kB-dependent pathway, an effect which is triggered specifically through TLR4.

## Supplementary Information

Below is the link to the electronic supplementary material.

Supplementary file1 (DOCX 161 kb)

## Data Availability

Data supporting the findings of this study are available from the corresponding author upon reasonable request.
