# Supplementary material for: Extracellular histones trigger oxidative stress-dependent induction of the NF-kB/CAM pathway via TLR4 in endothelial cells
Source: J Physiol Biochem. 2022 Dec 5;79(2):251–60. doi: 10.1007/s13105-022-00935-z (PMC10300178; doi:10.1007/s13105-022-00935-z)
Supplement: Supplementary file 1 — Supplementary file1 (DOCX 161 kb) [file 13105_2022_935_MOESM1_ESM.docx]

**Supplemental Figure 1**

**Supplemental Figure 1. (A)** HUVEC exposed to 50 µg/mL of histones for 4 h (n=3). Relative IL-1A, IL-1B, and IL-18 expression was determined by qRT-PCR. **(B)** HUVEC were transfected with NOX1 siRNA and negative control siRNA and exposed to 50 µg/mL of histones for 4 h (n=3). ROS production and relative VCAM1 expression were determined by DHE staining and qRT-PCR, respectively. **(C)** HUVEC exposed to 50 µg/mL of histones for 4 h (n=3). Relative MYD88 expression was determined by qRT-PCR. Data are expressed as mean ± SEM. ***P* < 0.01 *versus* 0 µg/mL of histones. ^##^*P* < 0.01 *versus* 50 µg/mL of histones.
